# Genetic polymorphisms within exon 3 of heat shock protein *90AA1* gene and its association with heat tolerance traits in Sahiwal cows

**DOI:** 10.14202/vetworld.2015.932-936

**Published:** 2015-07-31

**Authors:** Rakesh Kumar, I. D. Gupta, Archana Verma, Nishant Verma, M. R. Vineeth

**Affiliations:** Division of Dairy Cattle Breeding, Indian Council of Agricultural Research-National Dairy Research Institute, Karnal, Haryana, India

**Keywords:** heat stress, heat tolerance coefficient, *HSP90AA1*, polymorphisms, Sahiwal cattle

## Abstract

**Aim:**

The present study was undertaken to identify novel single nucleotide polymorphism (SNP) in Exon 3 of *HSP90AA1* gene and to analyze their association with respiration rate (RR) and rectal temperature (RT) in Sahiwal cows.

**Materials and Methods:**

The present study was carried out in Sahiwal cows (n=100) with the objectives to identify novel SNP in exon 3 of *HSP90AA1* gene and to explore the association with heat tolerance traits. CLUSTAL-W multiple sequence analysis was used to identify novel SNPs in exon 3 of *HSP90AA1* gene in Sahiwal cows. Gene and genotype frequencies of different genotypes were estimated by standard procedure POPGENE version 1.32 (University of Alberta, Canada). The significant effect of SNP variants on physiological parameters, e.g. RR and RT were analyzed using the General Linear model procedure of SAS Version 9.2.

**Results:**

The polymerase chain reaction product with the amplicon size of 450 bp was successfully amplified, covering exon 3 region of *HSP90AA1* gene in Sahiwal cows. On the basis of comparative sequence analysis of Sahiwal samples (n=100), transitional mutations were detected at locus A1209G as compared to *Bos taurus* (NCBI GenBank AC_000178.1). After chromatogram analysis, three genotypes AA, AG, and GG with respective frequencies of 0.23, 0.50, and 0.27 ascertained. RR and RT were recorded once during probable extreme hours in winter, spring, and summer seasons. It was revealed that significant difference (p<0.01) among genetic variants of *HSP90AA1* gene with heat tolerance trait was found in Sahiwal cattle. The homozygotic animals with AA genotype had lower heat tolerance coefficient (HTC) (1.78±0.04^a^), as compared to both AG and GG genotypes (1.85±0.03^b^ and 1.91±0.02^c^), respectively. The gene and genotype frequencies for the locus A1209G were ascertained.

**Conclusions:**

Novel SNP was found at the A1209G position showed all possible three genotypes (homozygous and heterozygous). Temperature humidity index has a highly significant association with RR, RT, and HTC in all the seasons. Perusal of results across different seasons showed the significant (p<0.01) difference in RR, RT, and HTC among winter, spring, and summer seasons. Genetic association with heat tolerance traits reveals their importance as a potential genetic marker for heat tolerance traits in Sahiwal cows.

## Introduction

Most part of India lies in a tropical region where temperature even goes up to 46°C during summer. Global warming has resulted in extensive climatic changes in tropical regions, resulting in increased heat stress in dairy animals of the region. Due to these generated pertinent heat stress to livestock, reduction of productivity with devastating economic consequences [1], decreased milk production [2], and a lower reproductive success rate [3] were seen. The loss of milk production due to heat stress in monetary terms amounts to a whopping Rs. 26616.2 million crore per year [4]. An increase of about 0.9°F in cows’ body temperature has been estimated to cause 12.8 % decline in conception rate in cattle [5]. Suitable breeding programs can help to achieve animal population that could cope with effects of heat stress [6]. Indigenous (*Bos indicus*) cattle survive and perform better under heat stress as compared to temperate breeds or their crossbreds [7,8] due to the high prevalence of heat shock protein (HSP) gene. Genetic differences in thermotolerance at the physiological and cellular levels are documented by a number of studies on *Bos indicus* and *Bos taurus* cattle breeds [9-11].

Cellular tolerance to heat stress is mediated by a family of proteins named as HSP. Among members of the HSP family, HSP70 and HSP90 are the most abundant proteins in eukaryotic cells. HSP90 act as important molecular chaperones that are constitutively expressed as a consequence of heat or stress induction [12]. Heat shock factor 1 is a transcription factor that is involved in the general maintenance and up-regulation of HSP90 protein expression [13]. Earlier study reported that HSP90 expression was increased due to heat stress in murine embryonic fibroblast cells [14], lung, heart, spleen, liver, and brain of human [15]. The “trait” heat tolerance is a quantitative trait [16,17]. There are two major isoforms of HSP90, which have arisen by gene duplication, HSP90α or *HSP90AA1* (inducible form), and HSP90β or HSP90AB1 (constitutive form). *HSP90AA1* gene in Deoni cattle (*Bos indicus*) has been found to be polymorphic and showed significant association with productive and reproductive parameters [18]. Many earlier studies have shown an association between single nucleotide polymorphisms (SNPs) at certain HSP genes and heat resistance [19,20]. HSP gene families have been widely discussed as candidate genes for heat resistance [21], and few studies have shown an association between SNPs at *HSP90AA1* genes and heat resistance in different species.

However, no report is available on *HSP90AA1* gene variants and their association with thermotolerance in Indian dairy cattle breeds. Keeping in view the importance of *HSP90AA1* gene, the present study was undertaken to determine the genetic polymorphism of exon 3 of *HSP90AA1* gene in Sahiwal breed of cattle and to associate the observed genetic polymorphisms with heat tolerance traits.

## Materials and Methods

### Ethical approval

The experimental plan of study was duly approved by Institution Animal Ethics Committee of National Dairy Research Institute, Karnal, Haryana, India

### Resource population

The experimental study was carried out in Sahiwal cows (n=100), maintained at Livestock research complex at National Dairy Research Institute, Karnal, located at 29.68°N latitude and 76.98°E longitude with altitude ranging from 235 to 252 meters above mean sea level.

### Experimental animals and DNA extraction

About 10 ml of blood was collected in EDTA coated vacutainer tube from each of the Sahiwal cows and stored at −20°C until DNA isolation. Genomic DNA was extracted from the blood samples using phenol - chloroform extraction method described by Sambrook and Russell [22] with minor modifications. The quality of DNA was checked by 1.5% agarose gel electrophoresis. Quality and quantity of DNA was also estimated by Biospec-nano spectrophotometer (Shimadzu co-operation, Japan). The ratio between OD_260_ and OD_280_ was observed for each sample. DNA sample with a ratio of 1.8 was considered good and taken for further analysis. The genomic DNA was diluted to a final concentration of 30 ng/µl, stored at −20°C.

### Physiological parameters recorded

Respiration rate (RR) and rectal temperature (RT) were recorded once daily for 3 days consecutively during probable extreme hours in winter, spring, and summer seasons and average was taken as final reading for association analysis. HTC was calculated based on a heat tolerance index developed by Benezra [23]. The formula is based on both RR and RT.

HTC: RR/23 + RT/38.33

Temperature humidity index (THI) measures the combined effects of ambient temperature and relative humidity to ascertain heat load intensity [24]. THI was calculated for all days in three seasons *viz*. winter (48.77), spring (64.86), and summer (92.62) during which physiological parameters were recorded and used in the association analysis as fixed variables. THI calculation using dry-bulb temperature (Db) and wet-bulb temperature (Wb) to estimate the magnitude of heat stress [25], were the most common compared to other methods.

THI = 0.72 (Wb + Db) + 40.6

Where, Wb and Db are wet- and dry-bulb temperatures in °C, respectively.

### Polymerase chain reaction (PCR) primers and amplifications

Primer was designed based on the bovine *HSP90AA1* gene sequence (NCBI GenBank AC_000178.1) using the Primer3 software. The sequence of primers, their respective nucleotide numbers, targeted region, and amplicon size are given in Table-1. The PCR reactions were carried out on a total of 25 µl volume containing template DNA of 3 µl (30 ng/µl), 1.0 µl of forward and reverse primer, PCR Master Mix (2x) (Fermentas) of 12.5 µl, and 8.5 µl of water. Amplification was performed in a Thermal cycler (MJ research and BioRad, T100). The thermal cycling conditions involved an initial denaturation at 95°C for 3 min, followed by 35 cycles with initial denaturation at 95°C for 30 s, annealing temperature of 55.7°C for 45 s, extension at 72°C for 1 min followed by a final extension at 72°C for 5 min. PCR program for both the primers was found to be same. PCR products were detected by electrophoresis on 2% agarose gel stained with ethidium bromide.

**Table-1:** Primer sequences, targeted region, and amplicon sizes of bovine *HSP90AA1* gene.

Primer set	Sequence (5’ -3’)	No. of base	Targeted region on gene (*HSP90AA1*)	Amplicon size (bp)
1.	F-GCGTCATCACGTGTCATCTT R-CCTCCTTTGGGGTTCCAGT	20 19	Exon 3 (826….1276)	450 bp

### Statistical analysis

The gene and genotypic frequencies of different genotypes were estimated by standard procedure POPGENE version 1.32 (University of Alberta, Canada) [26]. The association of SNP genotype with RT, RR, and heat tolerance coefficient (HTC) was analyzed using General Linear model procedure of SAS Version 9.2. The significant effect of SNP variants on physiological parameters was analyzed using the following model:

Y_ijk_ = µ + T_i_ +SNP1_j+_e_ijk_

Where,

Y_ijk_ = K^th^ observation on RR/RT/HTC of cows in i^th^ THI and j^th^ SNP1

µ= Overall mean

T_i_= Effect of ith THI (I = 48.77, 64.86, and 92.62)

SNP1_j_= Effect of j^th^ genotype of SNP1 A1209G (J = AA, AG, and GG)

## Results

### Analysis of sequence data

**Figure 1:**
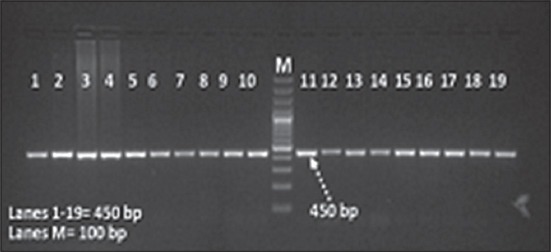
PCR amplification of exon 3 of *HSP90AA1* gene in Sahiwal cow (M=100bp)

The PCR product with the amplicon size of 450 bp was successfully amplified, covering exon 3 region of *HSP90AA1* gene in Sahiwal cows (Figure-1). The final sequence of the contig for Sahiwal cows were deduced from the raw sequences by using BioEdit software. ClustalW software, with a reference sequence of *Bos taurus* (NCBI GenBank AC_000178.1) was employed for determining the polymorphism in exon3 of Sahiwal cow.

### SNP detection of bovine *HSP90AA1* gene

**Figure 2:**
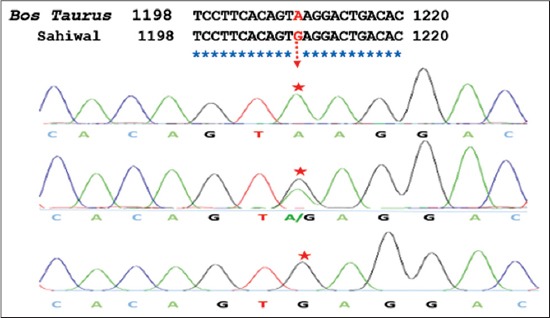
Chromatograph showing SNP at position 1209 A>G of *HSP90AA1* gene in Sahiwal cow

On the basis of comparative sequence analysis of Sahiwal samples (n=100), transitional mutations were detected at locus A1209G as compared to *Bos taurus* (NCBI GenBank AC_000178.1). After chromatogram analysis, three genotypes (AA, AG, and GG) were detected at an A1209G locus. Different chromatograms showing polymorphism (SNPs) as compared to *Bos taurus* has been depicted in Figure-2. The allelic and genotypic frequencies of *HSP90AA1* gene are given in Table-2.

**Table-2:** Gene and genotypic frequencies of the patterns/variants in Sahiwal cattle population.

*HSP90AA1* gene Loci	Genotypes	Genotypic frequencies	Gene frequencies
Exon 3	AA	0.23	A 0.48
	AG	0.50	
	GG	0.27	G 0.52

### Association of heat tolerance traits with novel SNP

For association study RR, RT, and HTC were taken as dependent variables. Relationship of each dependent variable with THI and novel SNP variants of *HSP90AA1* gene was analyzed.

Overall least square means of RR per minute, RT (°C), and HTC were found to be 19.75±0.42, 38.22±0.05, and 1.85±0.02, respectively (Table-3). RR per minute of each genotype were observed as AA (18.40±0.46^a^), AG (19.60±0.85^b^), and GG (21.18±0.64^c^) and RT (°C) of each genotype AA (37.91±0.12^a^), AG (38.32±0.10^b^), and GG (38.27±0.08^b^) were observed during winter, spring, and summer seasons. The corresponding values of HTC of each genotype, AA (1.78±0.04^a^), AG (1.85±0.03^b^), and GG (1.91±0.02^c^) were observed in respective seasons. Perusal of results across different seasons showed the highly significant (p<0.01) in RR, RT, and HTC between winter, spring, and summer seasons. Overall the results revealed an increasing trend in RR, RT, and HTC with the increase in THI. On the other hand, novel SNP found highly significant (p<0.01) associated with RR and HTC, but with RT, low significant (p<0.05) was observed.

**Table-3:** Least squares means of subclasses of different fixed effects for RR, RT, and HTC in Sahiwal cows.

Effect	Subclass	RR**	RT*	HTC**
Overall mean		19.54±0.42	38.17±0.05	1.85±0.01
THI	48.77	14.22±0.43^a^	37.92±0.05^a^	1.60±0.01^a^
	64.86	18.15±0.43^b^	38.19±0.06^b^	1.81±0.01^b^
	92.62	26.26±0.41^c^	38.40±0.06^b^	2.16±0.03^c^
A1209G	AA (23)	18.40±0.46^a^	37.91±0.12^a^	1.78±0.04^a^
	AG (50)	19.60±0.85^b^	38.32±0.10^b^	1.85±0.03^b^
	GG (27)	21.18±0.64^c^	38.27±0.08^b^	1.91±0.02^c^

RR=Respiration rate, RT=Rectal temperature, HTC=Heat tolerance coefficient, figures in parenthesis are number of animals; means with different superscripts within the column differ significantly

** (p<0.01,

* p<0.05)

## Discussion

The average RR value for locus A1209G of genotype AA (18.40±0.46^a^), AG (19.60±0.85^b^), and GG (21.18±0.64^c^) were highly significantly (p<0.01) associated from each other. Effect of RT on SNP locus A1209G of genotype AA (37.91±0.12^a^) significantly (p<0.05) differ with AG (38.32±0.10^b^) and GG (38.27±0.08^b^) genotype. Effect of HTC for genotype AA (1.78±0.04^a^), AG (1.85±0.03^b^), and GG (1.91±0.02^c^) differed highly significantly (p<0.01) from each other. The homozygotic animals with AA genotype had lower HTC (1.78±0.04^a^), as compared to both genotype AG and GG (1.85±0.03^b^ and 1.91±0.02^c^), respectively.

Our study indicated that Sahiwal cows of AA genotype had better thermotolerance than the other two genotypes (AG and GG) since increased respiration is an important thermoregulatory response to heat stress. It aids intemperance of excess body moisture in the expired air [27]. Higher HTC value indicated lower adaptability to heat stress in summer. In contrast, allele T at SNP g.4338>C of HSP90AB1 gene was found to be associated with HTC in Thai native cattle [28], Sahiwal, and Frieswal cattle in India [29,30]. Marcos-Carcavilla [31] reported a SNP located at position - 660 in the 5′flanking region of *HSP90AA1* was associated with different thermal conditions in sheep. The results of the present study were first time reported, so no earlier reports are available to compare the present findings.

## Conclusions

The study was carried out in Sahiwal cows with the objectives to identify novel SNP in the *HSP90AA1* gene and to analyze their association with heat tolerance trait. Novel SNP was found at A1209G position and all possible genotypes were observed THI has a significant association with RR, RT, and HTC in all the seasons. The genetic variants observed in *HSP90AA1* of exon 3 and their genetic association with heat tolerance traits reveals the importance of homozygotic AA genotype, which had been useful for genetic improvement of Sahiwal cow for heat tolerance traits and can also be utilized as a genetic marker to select appropriate animals for hot climatic conditions of tropics and sub-tropics.

## Authors’ Contributions

RK, IDG, and AV: Substantially contributed to design and plan of the study. RK and IDG: Drafted the manuscript, analyzed, and interpreted the results. NV and VMR: Helped in the analysis of data, drafted, and revised the manuscript. All authors read and approved the final manuscript.
